# Prescribing patterns of antimicrobials in the Internal Medicine Department of Ibrahim Malik Teaching Hospital in Khartoum, 2016

**DOI:** 10.11604/pamj.2019.34.89.17023

**Published:** 2019-10-15

**Authors:** Salma Nasr Abdalla, Bashir Alsiddig Yousef

**Affiliations:** 1 Department of Pharmacology, Faculty of Pharmacy, University of Khartoum, Sudan

**Keywords:** Antimicrobial drugs, prescribing patterns, prescribing indicators, antimicrobial resistance

## Abstract

**Introduction:**

Antimicrobials are among the most commonly prescribed therapeutic agents in hospitals. Irrational use of antimicrobials results in the development of antimicrobial resistance which could lead to life-threatening illnesses. Therefore, the assessment of antimicrobial prescribing and use is of utmost importance. This study aimed to examine the prescribing patterns of antimicrobials in the Internal Medicine Department of Ibrahim Malik Teaching Hospital in Khartoum, Sudan.

**Methods:**

A descriptive, cross-sectional study was conducted using World Health Organization (WHO) indicators for antimicrobial use in hospitals. Systematic random sampling was used to select 245 medical records from the 2613 medical records of patients admitted to the internal medicine department in 2016. Data were collected using a data collection form and a structured interview with the chief pharmacist in the hospital.

**Results:**

Of the 245 medical records examined, 201 (82%) patients were prescribed one or more antimicrobial drug. The average number of antimicrobials per patient was (2.1±1.1). The average duration of antimicrobial treatment was (4.9±3.8) days. The generic name was used in (35.6%) of antimicrobials, while (95.5%) of all antimicrobials were prescribed from the national essential medicines list. Overall, there were 421 courses of antimicrobials prescribed. The most frequently prescribed antimicrobials were ceftriaxone (131 courses) and metronidazole (89 courses). Among the documented infectious diseases, the most frequently encountered was pneumonia, followed by malaria. There was no drug and therapeutic committee, hospital formulary or essential medicines list, and standard treatment guidelines for infectious diseases in the hospital.

**Conclusion:**

The results of the study revealed a high percentage of antimicrobial use in the Internal Medicine Department. Multifaceted interventions are urgently needed to promote rational prescribing of antimicrobials.

## Introduction

Antimicrobial drugs (AMD) are the most commonly prescribed medications in outpatient and inpatient settings [1,2]. While the rate of AMDs use in developed countries is around 30% [3], in developing countries, it ranges between 35% to 60% [4]. The burden of lower respiratory tract infections, diarrheal diseases, and tuberculosis (TB) represented 30% of the top ten causes of deaths globally in 2016 [5]. Infectious diseases are one of the major causes of mortality in developing countries [6]. In Sudan, 52.8% of diseases burden is attributable to infectious diseases [7]. This global burden on public health and the widespread use of antimicrobials necessitate their optimal use to reserve their efficacy. The irrational use of medicines is represented in different forms including polypharmacy, inappropriate indication, use of unnecessarily expensive drugs, and inappropriate use of antimicrobials [8]. The adoption of essential medicines list (EML) is a cornerstone of the rational use of medicines that helps in promoting the cost-effective use of drugs [9] and improving the quality of prescribing where drug information, prescribing training, and medical audit focus on a certain list of medicines [10]. Another essential element in rational prescribing is the use of the generic name, which contributes to safe prescribing and dispensing [11] and ensures the release of clear and unambiguous medication order [12], as well as containment of expenditure for drugs prescription. This is because generics are priced much less than the brand drugs [13,14]. Antimicrobial resistance (AMR) is a global problem that challenges public health by increasing morbidity, mortality, adverse drug effects, as well as the cost of treatment. The emergence of AMR is related to the consumption of antimicrobials. This is noticed with the positive correlation between antibiotics resistance (ABR) and consumption of antibiotics [15]. The highest consumption of AMDs at the hospital level is associated with the highest rate of resistance [16]. To ensure optimal and rational use of antimicrobials, information about antimicrobial prescribing patterns is very important since it helps in suggesting measures and interventions that improve AMDs prescribing practice. This study was carried out to investigate the use, and prescribing patterns of antimicrobials by using selected prescribing indicators in the the Internal Medicine Department and to investigate the existence of EML or hospital formulary (HF) and standard treatment guidelines (STGs) for infectious diseases that regulate prescribing of antimicrobials in Ibrahim Malik Teaching Hospital in Khartoum, Sudan.

## Methods

The study was a descriptive, cross-sectional, hospital-based study. It was conducted in the Internal Medicine Department of Ibrahim Malik Teaching Hospital, which locates in Khartoum locality and provides health services to all age groups. The Medical Department is divided into 44-bed short-stay ward and 30-bed long-stay ward. A systematic random sampling technique was used to select the sample (n= 245, calculated using OpenEpi) [17] from the medical records of all patients (N=2613) who were admitted to the Internal Medicine Department during the study period (January first 2016- December 31 2016). As the medical records were arranged chronologically, the sample size was divided among the 12 months by proportion, based on the number of medical records in each month.

**Inclusion criteria:** the medical records of patients who were admitted to the Internal Medicine Department during the study period for whom at least one antimicrobial drug was prescribed were included in the study.

**Exclusion criteria:** patients who were prescribed only topical antimicrobial or were admitted for less than 48 hours were excluded from the sample.

Five prescribing indicators and two hospital indicators were used to achieve the objectives of the study. The WHO recommends these indicators in the manual “How to investigate antimicrobial use in hospitals: selected indicators” [1]. The prescribing indicators used in this study include the percentage of hospitalizations with one or more antimicrobials prescribed, the average number of antimicrobials prescribed per patient, percentage of antimicrobials prescribed by generic name, percentage of antimicrobials prescribed consistent with the EML and average duration of prescribed antimicrobial treatment. The hospital indicators include the existence of STGs for infectious diseases and existence of an approved HF list or EML. The data were collected by using two instruments adapted from the WHO [1]. The first instrument was a data collection form slightly modified to accommodate the objectives of the study. All data regarding the prescribing of AMDs were documented which include: the name of AMD, the diagnosis, the duration of treatment, the documentation of the dose, route of administration, frequency of administration, use of the generic name, use of AMD from EML and the request of culture. The final form was produced after performing a pilot test to refine the study instrument. The pilot data were excluded from the final results of the study. The second instrument was a questionnaire consisting of eight questions conducted with the chief pharmacist in Ibrahim Malik Teaching Hospital to obtain data regarding the hospital indictors. For the analysis of the medical conditions of patients who were prescribed AMD, the documented diagnosis (professional or medical) was classified firstly according to the International Classification of Diseases ICD-10 [18], into 8 groups of diseases, and then the indications for antimicrobial prescribing documented in the medical records were reported. When more than one diagnosis was documented for the patient, if it included infectious disease, only the infectious disease was reported. Data were entered into the Statistical Package for Social Sciences (SPSS, version 20) and Microsoft Excel 2010. Descriptive analysis was conducted. The chi-square test was used to examine the association between the number of AMDs prescribed and the age, comorbidity, length of hospital stay and diagnosis. For the statistical significance, a p-value (<0.05) was considered significant. The research protocol was approved by the Directorate of Research, Ministry of Health and Khartoum State. Only data relevant to the study were extracted from the patient medical records, identifiable data were never taken from the medical records or used in a way that harms the patient, so the privacy and confidentiality were maintained.

## Results

The interview with the chief pharmacist revealed that the hospital lack structures that regulate the prescribing of antimicrobials. There was no drug and therapeutic committee (DTC), HF or EML specifically designed to the hospital; accordingly, the national EML was used in this study. Also, there was no STGs for infectious diseases as separate or as a part of comprehensive treatment guidelines adopted by the hospital. A total of 201(82%) out of 245 medical records examined were enrolled in the study in which at least one AMD was prescribed. Table 1 shows the demographic and clinical characteristics of study patients. The mean age of the study patients was 53.9±20.4 years with the age group (>65) representing (39.4%). Among the 201 patients, males represented 116 (57.7%). The median length of hospital stay was 6 days. Infectious and parasitic diseases (35.3%), diseases of the circulatory system (23.4%), and diseases of the digestive system (10%) were the most common medical conditions for patients who were prescribed AMDs Table 1. Table 2 shows the prescribing indicators for antimicrobial use. The percentage of antimicrobial prescribing among the randomly selected sample was (201/245) (82%). A total of 421 courses of AMDs were prescribed with an average number of 2.1 ±1.1 AMD per patient. The generic name was used in (35.6%) of all AMDs prescribed. The description and frequencies of AMDs courses are summarized in Table 3. Of the 201 patients who received AMDs, (32.2%) received one AMD, (41.3%) two AMDs and (26.4%) three or more AMDs. Indication documentation for which the AMD was prescribed and the route of administration was omitted in (56%) and (35.1%) of the total courses prescribed respectively. The culture was requested in 12 (6%) out of the 201 medical records, with only one result documented. Under the chi-square test, the length of hospital stay (p <0.001) and the type of diagnosis (p = 0.02) were associated with the number of AMDs prescribed (Table 4). Among the 15 classes of AMDs prescribed cephalosporins (49.1%) and metronidazole (21.4%) were the most prescribed (Table 5). By agents, ceftriaxone and metronidazole were the most prescribed AMDs (Table 5). The most common infectious disease for which antimicrobials were prescribed was pneumonia followed by malaria, (viral hepatitis, sepsis), and urinary tract infections (UTI) as shown in Table 6.

**Table 1:** Demographic and clinical characteristics of study patients (N=201)

Patient characteristics	Values	Total
Age (mean±SD)	53.9±20.4	200^++^
**Age distribution n (%)**		
≤20^+^	13 (6.5)	
20-34	25 (12.4)	
35-49	37 (18.4)	
50-64	46 (22.9)	
≥65	79 (39.4)	
**Gender n (%)**		
Males	116 (57.7)	201
Females	85 (42.3)	
**Comorbidity n (%)**		
Absent	119 (59.2)	201
Present	82 (40.8)	
**Residence n (%)**		
In Khartoum	99 (49.3)	201
Out site Khartoum	92 (45.3)	
Missed data	11(5.5)	
**Length of hospital stay (median, IQR)**	6 (3-10)	201
≤ 6	115 (57.3)	
> 6	86 (42.7)	
**Final status of patients n (%)**		
Discharged	116 (57.7)	201
Referred^+++^	9 (4.5)	
Left against medical advice	3 (1.5)	
Escaped	11(5.5)	
Died	62(30.8)	
**ICD- diagnosis n (%)**		
Infectious and parasitic diseases	71 (35.3)	201
Diseases of circulatory system	47 (23.4)	
Diseases of digestive system	19 (9.5)	
Diseases of the respiratory system	13(6.5)	
Diseases of blood	13 (6.5)	
Neoplasms	10 (5.0)	
Diseases of nervous system	6 (3.0)	
Endocrine and metabolic diseases	4 (2.0)	
Multiple diagnosis(noninfectious)	6 (3.0)	
Missed	5(2.5)	
Others	7(3.5)	

+ Includes adults from 17-20

++ one missing data

+++ referred to another unit or hospital

**Table 2:** Prescribing indicators for antimicrobial use in the Internal Medicine Department (N=201)

Indicator	Values
Percentage of patients with one or more AMD prescribed	(82%)
Average number of AMD prescribed per patient (mean±SD)	2.1±1.1
Average duration of prescribed AMD treatment in days (mean±SD)	4.9±3.8
Percentage of AMD prescribed by generic name	(35.6%)
Percentage of AMD prescribed consistent with EML	(95.5%)

**Table 3:** Description and frequencies of antimicrobial drugs prescribed for the study patients

Title	n (%)
Total number of AMD courses for 201 patients	421
Patients prescribed 1 AMD	65 (32.3)
Patients prescribed 2 AMD	83 (41.3)
Patients prescribed ≥ 3 AMD	53 (26.4)
**Route of administration**	
Oral	50 (11.90)
Parenteral	371 (88.1)
**Indication for the antimicrobial in the medical records (N=201)**	
Documented	82 (40.8)
Not documented	119 (59.2)
**Request of culture and sensitivity test in the medical records (N=201)**	
Yes	12 (6)
No	189 (94)
**Dose**	
Documented	392 (93.1)
Not documented	29 (6.9)
**Route of administration**	
Documented	273 (64.9)
Not documented	148 (35.1)
**Frequency of administration**	
Documented	416 (98.8)
Not documented	5 (1.2)

**Table 4:** Factors associated with prescribing multiple antimicrobial drugs

Variable	Number of patients		Chi-square P value
	>2AMD(n=53)	≤ 2 AMD(n=148)	
**Age**			
>55years	24 (24.2%)	75 (57.8%)	0.50
≤ 55years	29 (28.4%)	73 (71.6%)	
**Comorbidity**			
Present	24 (29.3%)	58 (70.7%)	0.44
Absent	29 (24.4%)	90 (75.6%)	
**Length of hospital stay**			
> 6 days	38 (44.2%)	48(55.8%)	<0.001
≤ 6 days	15 (13.0%)	100 (87%)	
**Diagnosis**			
Infectious and parasitic diseases	28 (39.4%)	43 (60.6%)	0.02
Diseases of circulatory system	6 (12.7%)	41 (87.3%)	
Diseases of digestive system	7 (36.8%)	12 (63.2%)	
Diseases of blood	2 (18.2%)	11 (81.8%)	

**Table 5:** Frequencies of the antimicrobial drugs prescribed for the study patients by class and agents (N=421)

Antimicrobial	Courses of treatment (%)
**Chephalosporins**	207 (49.1)
Ceftriaxone	131
Ceftazidime	31
Cefuroxime	27
Cefotaxime	12
Cefipime	6
**Nitroimidazole**	90 (21.4)
Metronidazole	89
**Fluoroquinolones**	28 (6.7)
Ciprofloxacin	24
**Macrolides**	28 (6.7)
Clarithromycin	22
Azithromycin	6
**Antimalarials**	22 (5.2)
Quinine	11
Artemether	10
**Others**	46 (10.9)

**Table 6:** Frequencies of infectious diseases for which antimicrobials were prescribed

Infectious Disease	No of Patients
Pneumonia	19
Malaria	14
Viral hepatitis	10
Sepsis	10
UTI	9
Meningoencephalitis	8
Gastroenteritis	6
TB	4
Others	13

## Discussion

In this study, analysis of the demographic data showed that the majority (39.4%) of study patients were elders (>65 years). This may explain the high percentage of comorbidities (40.8%) among the study patients. In accordance with results reportedelsewhere [19-22], out of the total of 201 patients (57.7%) were male. Among the 245 medical records that were randomly selected, the percentage of patients who were prescribed one or more antimicrobial was 201 (82%), comparable to a Ugandan study [23] which reported (79%), and lower than results observed in Pakistan [24] (89%,91%), but higher than those reported in Nepal, [19] Nigeria, [22] and one study conducted in the medicine ward in Elobeid teaching hospital in Sudan, [25] which were (29.5%), (40%), and (58%) respectively. Although there is no standard value for this indicator, this value (82%) is alarming because as the use of the antimicrobials is increased the development of AMR will increase too [26]. This finding indicates the need for further studies to assess the appropriateness of antimicrobial use. The average number of antimicrobials per patient was 2.1 which was in accordance with the results in Nepal study, [27] but higher than the results reported by Elfaki [25] and Deshmukh *et al.* [28] In the absence of standard values for the prescribing indicators which should be developed according to the local patterns of diseases, policies and treatment guidelines; this value should be kept as low as possible. However, it might be considered acceptable in hospital settings where the cases are more complicated. During the hospital stay (26.4%) of patients received 3 or more AMDs which was higher than the result observed in a study from Nepal, [19] but lower than the finding from an Indian study [28] in which the percentages were (15%) and (50.5%) respectively. This study revealed a significant relationship between the number of antimicrobials prescribed and the length of hospital stay. Patients who spent longer duration (>6 days) in the hospital received more than two AMDs in comparison to those who spent less than 6 days. This finding is consistent with a study carried out in India [29]. This association might be explained by the high risk of acquiring nosocomial infections with more prolonged exposure to the hospital environment [30]. We also expect that patients with longer hospital stay are more likely to be undergoing invasive and surgical procedures which increase the risk of infections [31]. Another significant relationship was found between the number of AMDs and the type of diagnosis. Patients whose primary diagnosis was infectious disease or diseases of the digestive system were more likely to receive more than two AMDs compared to patients with other types of diagnosis. Thus more attention is needed to these groups of patients.

The adjustment of antimicrobial therapy, according to the culture and sensitivity test results, is important to optimize the course of treatment and decrease the selection pressure by using antimicrobial drugs with a narrower spectrum [32]. Almost all prescriptions were empiric. The specimens for culture were requested for only 12(6%) patients; this indicates that the multiple prescribing of antimicrobials (three or more) was not due to change in regimen according to the sensitivity test result, but might be due to frequent unjustified changes in the regimen. The average duration of antimicrobial treatment was 4.9 days. To minimize the development of AMR, the clinical guidelines should guide the duration of antimicrobial therapy [1]. Concerning the documentation of the dose and frequency of administration they were reported in (93.1%) and (98.8%) of the prescribed AMDs, respectively. However, the route of administration was poorly documented, only in (64.9%) of the 421 prescribed courses of AMDs. The documentation of these elements is essential for optimizing patient care since it ensures the release of clear medication order while avoiding incorrect dose, route of administration, and dose frequency. The generic name was used in (35.6%) of the prescribed AMDs; this is comparable to Elfaki [25] result which was (37%). However, it was lower than those reported in Nigeria [22] and Nepal [27]. This result is obviously far away from the standard value advocated by the WHO (100%). Although in Sudan, the generic drugs in the market are much less than the brand, the use of the generic name in prescribing should be encouraged to avoid dispensing errors and duplication of treatment. Almost all (95.5%) of antimicrobials were prescribed from the national EML. The documentation of indication is one of the most critical standards for good practice, [33] that decreases inappropriate prescribing of antimicrobials [34]. Among the 201 reviewed medical records, the indication was documented in only 82 (40.8%) records. In the remaining 119 (59.2%) cases, there is a possibility of concomitant infectious disease which was not documented in the patient record in addition to the primary diagnosis on admission. However, this low percentage of documentation questions the rationale behind prescribing the AMDs. In Australia, the last national antimicrobial prescribing survey (NAPS) report revealed that the indication for antimicrobial prescription was documented in (72.5%) of the total prescription which was considered below the best practice of (>95%) [35].

Cephalosporins are still one of the most prescribed antibiotics in hospitals due to their wide spectrum and safety. In the current study, cephalosporins were the most prescribed class of antimicrobials constituting (49.1%) of all prescribed courses of AMDs. This is similar to what was reported in a one-day antibiotic usage audit conducted in Soba university hospital in Khartoum in 2011, in which (76%) of all prescribed antibiotics were cephalosporins [36]. Other studies from Nigeria [21] and Uganda [23] showed similar results. The third-generation cephalosporins constituted the majority of the total courses of AMDs prescribed in this study. This raises a concern regarding its impact on the development of ABR because of the collateral damage caused by this class of antibiotics [37], which favors the propagation of resistant bacteria [38]. Ceftriaxone and metronidazole were the most prescribed antimicrobial drugs. They were most likely used for the treatment of aspiration pneumonia, which is one of the complications of a cerebrovascular accident, a condition that is most frequently encountered among the diseases of the circulatory system. At the study time, the hospital lacks the structures that regulate the prescribing of antimicrobials like DTC, HF or EML, and STGs for infectious diseases. These structures have a leading role in the promotion of rational prescribing and reflect the commitment of the hospital towards the high quality of patient care [1]. One of the limitations of this study is that the findings cannot be generalized to other hospitals in Khartoum state. Also, the study did not assess the appropriateness of antimicrobial use which should be addressed by other studies in the future.

## Conclusion

The findings of this study showed a high percentage of antimicrobials prescribing among the random sample studied. Most of the antimicrobials were prescribed from the national EML. However, prescribing using the generic name was a matter of concern. The study revealed a problem of suboptimal documentation of route of administration and the indication for antimicrobials prescribed, both of them compromise the rational prescribing of antimicrobials. The third-generation cephalosporins were the most commonly prescribed class which may contribute to the emergence of AMR. The empiric therapy predominated in this study, which was indicated by a very low request of culture. Finally, the study revealed the lack of structures that regulate the prescribing of antimicrobials and promote its rational use in the hospital, like DTC, HF or EML, and STGs for infectious diseases. Several measures and strategies are needed to promote rational prescribing of AMDs like the introduction of the antimicrobial stewardship program, implementation of continuous educational programs for prescribers about rational prescribing of antimicrobials, as well as continuous supervision, audit and feedback.

### What is known about this topic

- Antimicrobial drugs are one of the most prescribed medicines;
- The rational use of antimicrobial drugs includes prescribing when only indicated, use of the generic name, prescribing from the EML or HF, and adoption of STGs.

### What this study adds

- Antimicrobial drugs were highly prescribed in the internal medicine department;
- The indication and route of administration were poorly documented;
- The hospital lacks measures and structures that regulate the prescribing of antimicrobials like DTC, HF or EML and STGs for infectious diseases.

## Competing interests

The authors declare no competing interests.
